# COVID-19-Triggered Miller Fisher Syndrome Masking Leptomeningeal Progression of Merkel Cell Carcinoma: A Fatal Diagnostic Dilemma

**DOI:** 10.7759/cureus.109932

**Published:** 2026-05-30

**Authors:** Oscar Diaz, Daniela Prado Escobar, Kian Memari, Ana Villaverde Brizuela, Alexander Kuss, Skarlet Garcia, Kevin Sande

**Affiliations:** 1 Internal Medicine, Palmetto General Hospital, Hialeah, USA; 2 Internal Medicine, Nova Southeastern University Dr. Kiran C. Patel College of Osteopathic Medicine, Clearwater, USA; 3 Family Medicine, Palmetto General Hospital, Hialeah, USA; 4 Medical Student, American University of Antigua, Miami, USA; 5 Internal Medicine, Universidad de Los Andes, Merida, VEN

**Keywords:** covid-19, guillain-barré syndrome, ivig, leptomeningeal carcinomatosis, merkel cell carcinoma, miller fisher syndrome, ophthalmoplegia, paraneoplastic syndrome

## Abstract

Miller Fisher syndrome (MFS) is a rare variant of Guillain-Barré syndrome (GBS) classically characterized by the triad of ophthalmoplegia, ataxia, and areflexia. Its association with viral triggers, including SARS-CoV-2, has been increasingly recognized. We present a complex case of a 72-year-old male with metastatic Merkel cell carcinoma undergoing chemotherapy who presented in March 2026 with progressive weakness and near-syncope in the setting of severe anemia and COVID-19 infection. During hospitalization, he developed ophthalmoplegia, areflexia, and ataxia, raising concern for Miller Fisher syndrome. Despite early initiation of intravenous immunoglobulin (IVIG), the patient experienced rapid neurological deterioration with bulbar involvement, leading to respiratory failure requiring mechanical ventilation. This case highlights the diagnostic challenges and clinical complexity of MFS in the setting of malignancy and concurrent infection, emphasizing the importance of early recognition and management of neuromuscular complications in high-risk patients.

## Introduction

Miller Fisher syndrome (MFS) is a rare immune-mediated neuropathy classically defined by the triad of ophthalmoplegia, ataxia, and areflexia, and is considered a variant within the Guillain-Barré syndrome (GBS) spectrum [1]. It accounts for approximately 1-5% of GBS cases in Western populations and is strongly associated with anti-GQ1b antibodies, which are present in up to 90% of cases [2]. The pathogenesis is believed to involve molecular mimicry, in which antibodies generated against infectious agents cross-react with peripheral nerve components, particularly those affecting cranial nerves [3].

Miller Fisher syndrome is most commonly preceded by infections such as *Campylobacter jejuni*, influenza, or other viral illnesses; however, emerging evidence has demonstrated an association with SARS-CoV-2 infection [4]. Additionally, paraneoplastic neurological syndromes, though rare, can mimic or precipitate immune-mediated neuropathies, particularly in patients with underlying malignancies [5].

Merkel cell carcinoma (MCC) is an aggressive neuroendocrine skin cancer associated with immunosuppression and poor prognosis in advanced stages [6]. Neurological complications in patients with MCC may arise from paraneoplastic processes, direct metastasis, or treatment-related toxicity, complicating diagnostic evaluation.

We present a diagnostically challenging case of suspected Miller Fisher syndrome in a patient with metastatic Merkel cell carcinoma and concurrent COVID-19 infection, illustrating the interplay between infection, malignancy, and immune-mediated neurological disease.

## Case presentation

A 72-year-old male with a past medical history of atrial fibrillation on apixaban, type 2 diabetes mellitus, hypertension, peripheral neuropathy, and metastatic Merkel cell carcinoma on chemotherapy presented in March 2026 with progressive generalized weakness and near-syncope.

Initial evaluation revealed severe normocytic anemia with hemoglobin of 7.3 g/dL, and SARS-CoV-2 polymerase chain reaction testing was positive. The patient received packed red blood cell transfusion with initial hemodynamic stabilization. Initial laboratory values are summarized in Table 1.

**Table 1:** Pertinent Laboratory Findings on Admission g/dL: grams per deciliter; µL: microliter; fL: femtoliter; mmol/L: millimoles per liter; mg/dL: milligrams per deciliter; U/L: units per liter; mg/L: milligrams per liter; SARS-CoV-2: severe acute respiratory syndrome coronavirus 2

Test	Observed value	Reference range
White blood cell count (×10³/µL)	6.9	4.5-11.0
Hemoglobin (g/dL)	7.3	13.5-17.5
Hematocrit (%)	22.1	41.0-53.0
Mean corpuscular volume (fL)	88	80-100
Platelet count (×10³/µL)	176	150-450
Sodium (mmol/L)	136	135-145
Potassium (mmol/L)	4.1	3.5-5.1
Blood urea nitrogen (mg/dL)	21	7-20
Creatinine (mg/dL)	0.96	0.6-1.3
Glucose (mg/dL)	162	70-110
Aspartate aminotransferase (U/L)	34	10-40
Alanine aminotransferase (U/L)	28	7-56
C-reactive protein (mg/L)	42	<10
SARS-CoV-2 polymerase chain reaction	Positive	Negative

Despite correction of anemia, the patient developed progressive neurologic symptoms, including diplopia, ptosis, gait instability, and worsening generalized weakness. Neurologic examination demonstrated bilateral ophthalmoplegia with lateral rectus involvement, right-sided ptosis, anisocoria, areflexia, mild dysmetria, and gait ataxia. Brain computed tomography and magnetic resonance imaging showed no acute infarct, hemorrhage, or mass effect.

Given the combination of ophthalmoplegia, ataxia, and areflexia, MFS was strongly suspected. Serum anti-GQ1b antibody testing was considered but not obtained because it was not available in-house and would have required send-out testing with delayed turnaround. Because the patient was rapidly deteriorating and antibody results would not have changed immediate management, treatment was not delayed. Lumbar puncture was recommended to evaluate for albuminocytologic dissociation, infection, malignant cells, and leptomeningeal disease; however, the patient declined the procedure. Consequently, cerebrospinal fluid analysis could not be performed, limiting the ability to definitively distinguish MFS from competing diagnoses such as leptomeningeal carcinomatosis or paraneoplastic neurologic disease.

Empiric intravenous immunoglobulin was initiated at 0.4 g/kg/day for five days. The patient was also treated for COVID-19 infection with remdesivir. During hospitalization, he developed worsening ptosis, decreased level of consciousness, dysphagia, and bulbar weakness. He subsequently developed acute hypoxic respiratory failure requiring emergent intubation and transfer to the intensive care unit. Aspiration pneumonia was suspected and treated with broad-spectrum antibiotics. The patient’s clinical progression and key interventions throughout hospitalization are summarized in Table 2.

**Table 2:** Timeline of clinical progression from initial presentation to respiratory failure Hgb: hemoglobin; SARS-CoV-2: severe acute respiratory syndrome coronavirus 2; IVIG: intravenous immunoglobulin; g/kg/day: grams per kilogram per day; ICU: intensive care unit

Timepoint	Clinical course
Day 1 (Admission)	Presentation with generalized weakness, near-syncope, and severe normocytic anemia (Hgb 7.3 g/dL); SARS-CoV-2 positive. Transfusion initiated.
Day 2–3	Development of neurological symptoms, including diplopia, bilateral ophthalmoplegia with lateral rectus involvement, right-sided ptosis, and gait instability. Neurological examination revealed areflexia and dysmetria. Brain imaging negative for acute intracranial pathology.
Day 3	Clinical diagnosis of suspected Miller Fisher syndrome. IVIG therapy initiated (0.4 g/kg/day).
Day 4–5	Progressive neurological decline with worsening cranial nerve involvement and decreased level of consciousness.
Day 5+	Progressive dysphagia and bulbar weakness with acute hypoxic respiratory failure requiring emergent intubation and mechanical ventilation.

The clinical picture remained diagnostically complex because of the patient’s metastatic MCC and concern for leptomeningeal involvement. Although MFS was favored based on the classic neurologic triad and temporal association with SARS-CoV-2 infection, alternative or concurrent etiologies included paraneoplastic neuropathy, leptomeningeal metastatic disease, chemotherapy-related neurotoxicity, COVID-19-associated encephalopathy, aspiration pneumonia, metabolic encephalopathy, and critical illness neuropathy.

Despite completion of intravenous immunoglobulin therapy, the patient remained critically ill with poor neurologic recovery. Given metastatic malignancy, respiratory failure, and poor overall prognosis, goals-of-care discussions were held with the family.

## Discussion

Miller Fisher syndrome represents a distinct clinical entity within the Guillain-Barré spectrum, characterized by cranial nerve involvement and relatively preserved limb strength compared to classic GBS [1]. The diagnosis is primarily clinical, supported by the presence of anti-GQ1b antibodies and cerebrospinal fluid findings of albuminocytologic dissociation, although these may not always be obtainable in critically ill or non-consenting patients [2].

This case highlights a diagnostically complex scenario in which multiple potential etiologies for neurologic decline coexisted. The patient’s presentation fulfilled the classic triad of MFS; however, alternative diagnoses, including leptomeningeal metastatic disease, paraneoplastic neuropathy, chemotherapy-related neurotoxicity, COVID-19-associated encephalopathy, metabolic encephalopathy, and critical illness neuropathy, remained important considerations. Paraneoplastic neurological syndromes are well described in association with malignancies and can mimic immune-mediated neuropathies through antibody-mediated neuronal injury [5].

The absence of cerebrospinal fluid analysis and anti-GQ1b antibody testing significantly limited diagnostic certainty. Cerebrospinal fluid studies would have helped evaluate for albuminocytologic dissociation, central nervous system infection, malignant cells, and inflammatory processes, while anti-GQ1b antibody positivity would have strengthened support for MFS. Nonetheless, given the patient’s rapidly progressive neurologic deterioration and development of bulbar symptoms, empiric treatment with IVIG was clinically justified despite incomplete confirmatory evaluation.

The association between COVID-19 and Guillain-Barré spectrum disorders has been increasingly reported, with proposed mechanisms including immune dysregulation and molecular mimicry [4]. In this case, SARS-CoV-2 infection likely served as a precipitating factor in a patient already predisposed due to underlying malignancy and immunosuppression.

Merkel cell carcinoma further complicates the clinical picture. MCC is associated with immune dysfunction and has been linked to paraneoplastic syndromes, although neurological manifestations are uncommon [6]. Additionally, chemotherapy-related neurotoxicity and potential leptomeningeal spread may contribute to neurological decline.

The rapid progression to respiratory failure in this patient underscores a critical aspect of GBS-spectrum disorders: the risk of neuromuscular respiratory compromise. Up to 30% of patients with GBS require mechanical ventilation, and early recognition of bulbar involvement is essential for timely intervention [7].

Management of MFS typically involves supportive care and immunotherapy with IVIG or plasmapheresis. While most patients experience favorable outcomes, the presence of comorbid conditions such as malignancy, infection, and advanced age is associated with poorer prognosis [8].

This case emphasizes the importance of maintaining a broad differential diagnosis in patients with complex medical histories and highlights the need for early empiric treatment when clinical suspicion for MFS is high, even in the absence of confirmatory testing.

## Conclusions

This case illustrates a diagnostically challenging presentation of suspected Miller Fisher syndrome occurring in the setting of SARS-CoV-2 infection and metastatic Merkel cell carcinoma. The classic triad of ophthalmoplegia, ataxia, and areflexia strongly supported MFS, but concern for leptomeningeal progression, paraneoplastic neurologic disease, chemotherapy-related neurotoxicity, infection, and metabolic encephalopathy created substantial diagnostic overlap. The absence of cerebrospinal fluid studies and anti-GQ1b antibody testing limited confirmatory evaluation and reduced diagnostic certainty. Nevertheless, the patient’s rapidly progressive neurologic decline and bulbar involvement warranted early empiric immunotherapy.

Clinicians should maintain a broad differential diagnosis when GBS-spectrum findings occur in patients with active malignancy and concurrent COVID-19 infection. Early empiric immunotherapy, multidisciplinary evaluation, and proactive monitoring for bulbar and respiratory compromise are essential, particularly in high-risk patients with advanced cancer or concurrent infection.
